# Functional analysis of type VII secretion system links to host immune evasion mechanism in *Mycobacterium tuberculosis*

**DOI:** 10.3389/fcimb.2026.1797994

**Published:** 2026-04-07

**Authors:** Karthikeyan Sundaram, Sridhar Rathinam, Ramalingam Bethunaickan, Uma Devi Ranganathan, Venkataraman Prabhu, Madhavan Dhanapal

**Affiliations:** 1 Department of Herbal Pharmacology and Environmental Sustainability, Chettinad Hospital and Research Institute, Chettinad Academy of Research and Education, Kelambakkam, Kanchipuram, Tamilnadu, India; 2 Chettinad Hospital and Research Institute, Chettinad Academy of Research and Education, Kelambakkam, Kanchipuram, Tamilnadu, India; 3 Department of Immunology, Indian Council of Medical Research (ICMR)- National institute for Research in Tuberculosis, Chennai, Tamilnadu, India; 4 Division of Medical Research, SRM Medical College Hospital and Research Centre, Kattankulathur, Chennai, Tamilnadu, India

**Keywords:** autophagy, ESAT 6 secretion system, immune evasion, mycobacterium tuberculosis, T7SS, tuberculosis

## Abstract

*Mycobacterium tuberculosis* causes tuberculosis, an infectious disease; this acid-fast bacillus has various functions that enable it to survive within the host. Importantly, the type VII secretion system plays a vital role in host immune evasion. However, the early secretory antigenic target secretion system (ESX) component is crucial for mycobacteria survival, plays a significant role in bypassing the host immune response, and is linked to the prognosis of the disease. The review aims to analyze the ESX-associated genes’ functions in defence mechanisms against host immune response. There are five types of ESX, with the ESX-1 effectors consisting of the heterodimers *EsxA/*ESAT-6 and *EsxB*/CFP-10. The precise membranolytic role of *EsxA* remains unclear; however, mycobacterial mutants deficient in *EsxA* show reduced membrane lytic activity and lack the ability to perforate phagosomes. ESX-5 substrates, such as glycine-rich and repetitive *PE_PGRS* proteins, are associated with immune evasion and pathogenicity. ESX-5 releases a substantial amount of PE and PPE proteins, along with various other immune-modulating substrates. In addition, ESX-3 facilitates iron acquisition through mycobactin and regulates metal homeostasis. ESX 4 has been studied in two fast-growing mycobacterial species: *M. abscessus* and *M. smegmatis*. Notably, conjugal DNA transfer in the recipient strain of *M. smegmatis* requires ESX-4. Therefore, the type VII secretion system of ESX-associated genes plays a crucial role in bacterial survival and action against autophagosome-lysosome fusion. Thus, studying this system will explore the effects of specific antigenic structures and their relationships with autophagy and mycobacterial self-defense mechanisms.

## 1 Introduction

Tuberculosis (TB) is an infectious disease transmitted via airborne droplet nuclei. The causative agent is *Mycobacterium tuberculosis* (MTB). A single infectious agent is linked to increased mortality rates in humans (World Health Organization, 2024). MTB from disintegrating granulomas is aerosolized during coughing, facilitating the dissemination of the pathogen from the airways to the lung interstitium in persistent MTB infections. MTB infiltrates the lung interstitium through an unidentified mechanism, in contrast to other bacterial infections (Cohen et al., 2018). Besides that, mycobacteria utilize various mechanisms to persist within the host, relying on multiple biological systems. The pathophysiology of disease is dependent on the type VII secretion system. The early secretory antigenic target secretion system-1 (ESX-1) performs multiple functions, with a primary role in evading the host immune response (Cohen et al., 2018; Huang et al., 2018). Understanding the pathogenesis of MTB is crucial; however, the pathogenicity of MTB is contingent upon the type VII secretion system, specifically the ESX systems, which consist of ESX-1 – 2 – 3–4 and - 5. Importantly, ESX-1, 3, and 5 were studied well; however, they participate in phagosome escape, inhibit phagolysosome maturation, and facilitate the recruitment of innate immune cells, acquisition of iron by mycobactin, and uptake of calcium linked to ESX-3 and *PE_PPE* proteins linked to ESX-5. The region of difference 1 (RD1), which encodes the majority of the ESX-1 machinery, is lacking in the historical vaccine strain Bacillus Calmette-Guerin (BCG) (Zheng et al., 2025). Recent studies indicate that MTB adapts to stress through the phase change of homopolymers (Safi et al., 2019; Luna et al., 2025). Notably, the initial identification of this process in MTB was noted at the *glpK* gene, which encodes the glycerol-3 kinase necessary for assimilation. The seven-base-pair poly-G sequences of these genes demonstrate considerable mutagenicity in mycobacteria (Smith et al., 2022; Dupuy et al., 2023). Alongside *glpK*, phylogenomic investigations of a significant collection of clinical isolates indicated high-frequency indels in 44 homopolymeric tracts, implying positive selection. Numerous varied homopolymeric tracts were identified as being linked to the ESX-1 type VII secretion system, a crucial virulence element of MTB that performs various roles throughout infection (Vargas et al., 2023). Therefore, the review aims to analyze the ESX-associated genes functions in defence mechanisms against host immune response.

## 2 Type VII secretion system – ESXs

Pathogenic mycobacteria utilize unique type-7 protein secretion systems (T7SS) to excrete diverse virulence effector proteins (Dumas et al., 2016). The espACD operon is essential for the pathogenicity of MTB, and the esx-1 locus encodes the ESX-1 secretion system. The five systems (ESX-1 to ESX-5) are found in MTB. An oligomeric complex of five conserved proteins (*EccCa1, EccB1, EccD1, EccCb1*, and *EccE1*) is expected to constitute the ESX-1 secretion system, analogous to the structural examination of ESX systems in other mycobacteria, including ESX-3 and ESX-5. The five-component translocon core, in conjunction with *PE35, EccA1, EspD, PPE68, EspK, EspI, EspL*, and *MycP1* proteins, is hypothesized to translocate the ESX-1 substrate proteins *EspA, EspB, EspC, EsxA*, and *EsxB* in MTB in an ATP-dependent mechanism (Elliott et al., 2019; Drever et al., 2021).

### 2.1 ESX-1 (ESAT-6 secretion system - 1)

ESX-1 is a type VII secretion system present in MTB, essential for mycobacterial survival within macrophages. The interruption of the secretory route in the vaccination strain leads to BCG attenuation. An *EsxA/ESAT-6* and *EsxB/CFP-10* heterodimer functions as an ESX-1 effector and *EsxA* disrupts phagosomes. Recent studies indicate that mycobacterial mutants lacking *EsxA* exhibit reduced membrane lytic activity and fail to perforate the phagosome; nonetheless, its direct membranolytic effect remains unclear. The cytoplasmic membrane (CM) comprises a complex of conserved ESX-1 components (Eccs). ESX-1 membrane complexes recognize ESX-1 substrates and facilitate energy and pore formation for cytoplasmic membrane export. The protein substrates permeate the periplasm and mycolate outer membrane via an unidentified mechanism (Conrad et al., 2017; Sanchez et al., 2020).

**Figure 1:**
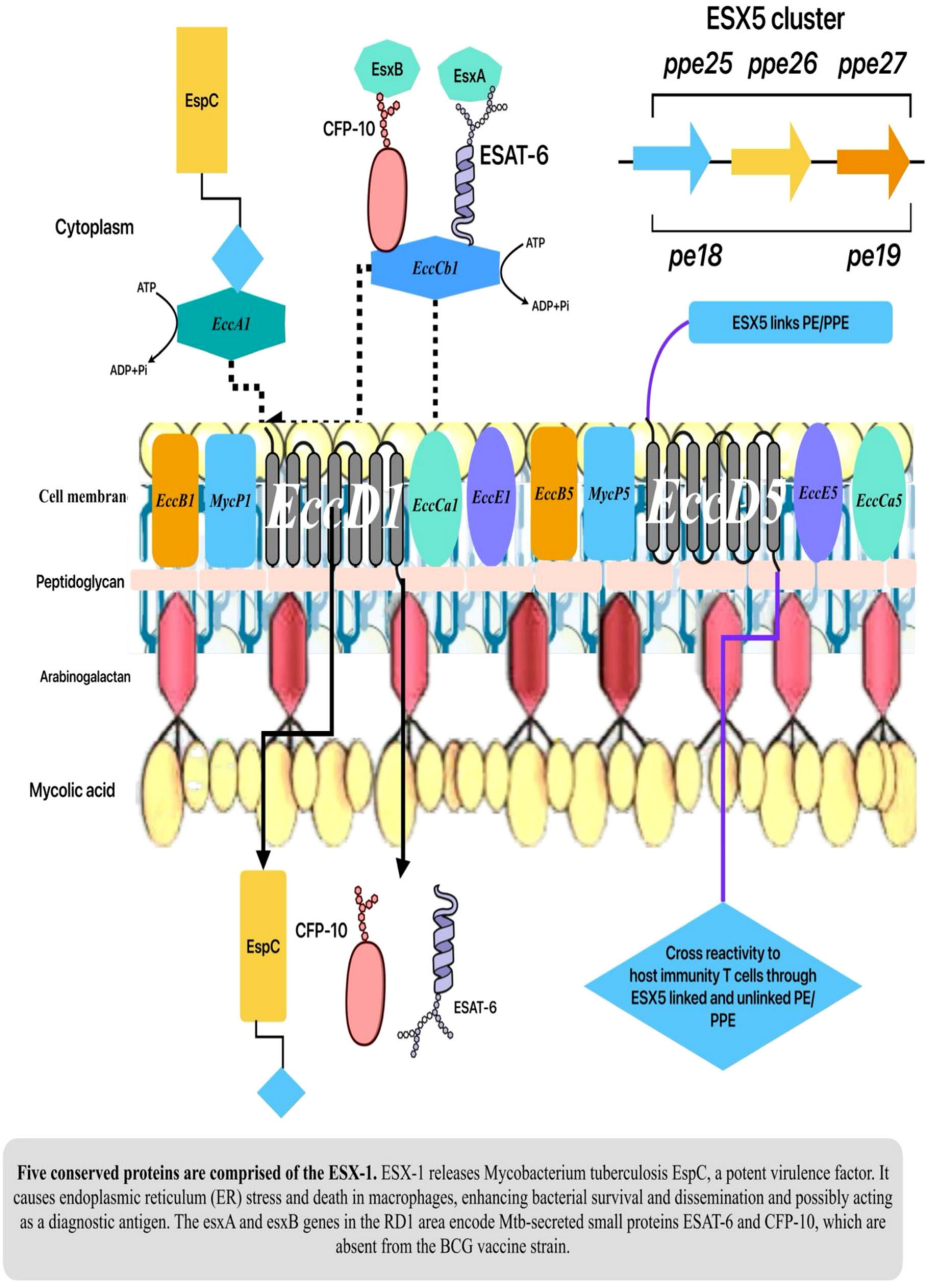
Mechanism of ESX-1 in MTB pathogenesis.

The pathogenicity of MTB is contingent upon the components of ESX-1 inside Region of Difference 1 (RD1). Notably, individuals with type 2 diabetes and nondiabetics who had BCG vaccination with ESX-1 exhibited an increased presence of CD11c+ dendritic cells and CD11b+ macrophages in their pulmonary tissue. Besides, T cell proliferation and activation need antigen-presenting cells. BCG: RD1 markedly enhances dendritic cell synthesis of proinflammatory cytokines IL-1β and TNF-α, resulting in heightened proliferation of IFNγ+ T cells (Etna et al., 2015; Sathkumara et al., 2020). Furthermore, other ESX-1 substrates with virulence-enhancing properties, including as *EspA, EspE*, and *EspF*, together with oligomers of *EspB* and *EspC*, are regarded as possible mycobacterial factors. These components may contribute to a suggested extracellular secretory complex that enables contact-dependent lysis (**Figure 1**) (Lou et al., 2017; Gijsbers et al., 2021; Osman et al., 2022).

The EsxAM93T variant demonstrated that moderate *EsxA* secretion sufficed for ESX-1-mediated lytic activity and promoted reduced secretion of *EsxA* and *EsxB* from *M. marinum*. Osman et al., who initially investigated the EsxAM83I and EsxAM93T protein variants, posited that both resulted in diminished virulence while preserving the secretion of ESAT-6 (*EsxA*) and CFP-10 (*EsxB*). Also, an EccCb1 ATPase facilitates the translocation of ESX-1 substrates from the mycobacterial cytoplasm by *EsxA* and *EsxB* (Osman et al., 2022; Prest et al., 2024). The bacterial cell secretes PE and PPE proteins both *in vitro* and within the host phagocyte, which are found on the surface of mycobacterial cells. ESX systems release various PE and PPE proteins that play a role in bacterial persistence and interaction with the host immune response (Bosserman et al., 2019). Also, the ESX-1 Type VII secretion system promotes phagosome permeabilization to enhance Mtb-host cytosolic pathway connections. ESX-1 is also needed for phagosome maturation arrest (PMA) (Xander et al., 2024). These traits suggest that the ESX-1 system permeabilizes phagosomes to let effectors into macrophage cytoplasm. The mycobacterial lipid phthiocerol dimycocerosate (PDIM), necessary for PMA, enhances ESX-1’s membrane permeabilization effects (Augenstreich et al., 2017; Quigley et al., 2017; Xander et al., 2024).

In this context, a significant bacillary burden may be required to generate sufficient ESX-1 complexes for the transport of MTB antigens across the phagosomal membrane. Cytosolic surveillance mechanisms, such as STING, identify cytosolic MTB and may improve MTB antigen cross-presentation (Mott et al., 2023). On the other hand, the *M. microti* ATCC 35782 and the three clinical strains studied all had a complete *plcABC* gene cluster but have widely diverse degrees of virulence, thus going against the concept that *plc* genes affect MTBc virulence. In addition to *plcABC*, this RD5mic locus contains *ppe38, ppe71, esxX*, and *esxY* (Orgeur et al., 2021). The complementation of BCG with cosmid pRD1-2F9, which encodes ESX-1 from MTB, typically results in a notable phenotypic alteration. However, the comparable transfer efficiencies observed between the recombinant and parental BCG strains indicate that ESX-1 does not play a role in the transfer process of tubercle bacilli. The authors concluded that the functional equivalence of MTB and *M. smegmatis* ESX-1 regions was demonstrated by the variable complementation of pRD1-2F9 in various hyperconjugative ESX-1 knockout mutants (Madacki et al., 2021).

The ESX-1 is essential for host cell infection, bacterial dissemination, and evasion of macrophages, but not for colonization in axenic culture (**Table 1**) (Theobald et al., 2021). Notably, infected host cells undergo necrotic cell death and phagosomal escape due to substrates produced by *EsxA*. The secretion machinery is irregularly regulated without an ESX-1 autoregulatory feedback mechanism that monitors intracellular substrate levels. Gram-negative bacteria’s type 3 secretion (T3SS) system’s intrinsic regulatory mechanism links substrate or regulatory protein secretion to variant T3SS component transcription. S3_100 and S3_106 differently enhanced ethionamide effectiveness. S3_106 prevented MTB-induced cell death and *EsxA* secretion; however, *EthA* and other monooxygenases did not upregulate over the EC50 (Theobald et al., 2021; Gries et al., 2024). In addition, recent study indicates that mutants and genetic tools can elucidate the location and function of *EccE1*, an unidentified ESX-1 membrane protein, in MTB. Although, this study demonstrates that *EccE1* is essential for macrophage lysis, a critical process in MTB pathogenesis, its absence does not influence antibiotic susceptibility or bacterial growth *in vitro* (Soler-Arnedo et al., 2020).

**Table 1:** The functional role of ESX-1, and significant biomarkers action in MTBc.

Study	Biological markers	Outcomes	Technical platform
(Cohen et al., 2018)	IL-1	IL-1 production and Inflammasome activation necessitate Mtb ESX-1-mediated phagosome bacterial escape. AM translocation to the interstitium precedes bacterial myeloid cell spread.	mice infected with the H37Rv:ΔRD1 (ΔRD1) strain of Mtb
(Zheng et al., 2025)	EccD1	In infected mice, MTB lacking EccD1 produced and reduced CD11chi-MNC, CD11clo-MNC, and neutrophils, similar to RD1 elimination.	mice infected with H37RvΔRD1, flow cytometry
(Luna et al., 2025)	EspR	The genes most downregulated were EspR-binding lipid esterases such lipU, lipL, and lipF. This mutation did not significantly dysregulate espR mRNA, which is interesting.	RNAseq analysis
(Drever et al., 2021)	EspB, Esx A, and Esx B INH	MTB growth is decreased to 8% with 0.182 μM INH, whereas EspB, EsxA, and EsxB abundance in CF and CL fractions remains unaltered. Additional INH raised the CF's Ag85 levels.	Sauton's medium with 0.091 μM (0.012 μg/mL) INH
(Sanchez et al., 2020)	MMAR_5438 binds whiB6	numerous proteins bound the whiB6 promoter bait better than nonspecific DNA. MMAR_5438 binds whiB6 promoter bait ≥64.0-fold ± 0.4-fold better than rpoA bait. Rename MMAR_5438 "espM." to match ESX-1.	liquid chromatograph- mass spectrometry (LC-MS/MS)
(Sathkumara et al., 2020)	BCG strains. CD4+ and CD8+ T cells	costimulatory marker expression of mLN CD11c+ DCs 24 hours after in vivo LPS instillation.	flow cytometry-based cellular phenotyping
(Osman et al., 2022)	Mm-eccATn	Mm-eccA1::Tn was lacking for RBC lysis. M. marinum-eccA1:: Tn could damage phagosomal membranes using galectin-8 labeling.	Immuno blot, immunofluorescence microscopy
(Prest et al., 2024)	EspE	M. marinum (WT) strain produced espE transcript, while ΔespE strain did not. Targeted mutations and the pMEFG wild-type plasmid restored espE transcript levels.	reverse transcriptase quantitative PCR (RT-qPCR)
(Xander et al., 2024)	ΔsapM, SatS	both ΔsapM Mtb and BCG mutants had intact SatS levels, demonstrating that the sapM deletion did not affect downstream satS expression.	Immunoblot analysis

### 2.2 ESX-2 (ESAT-6 secretion system - 1)

ESX-2 includes the complete ESX components, such as espG and espI, arranged in an operonic structure, while ESX-5 consists solely of espG along with several copies of PE and PPE, a ferredoxin, and a cyp143 gene. Despite the correlation between ESX-2 duplication and reduced growth along with adverse traits, it is lacking in multiple species (*M. leprae, M. marinum, M. ulcerans subsp. liflandii*, and *M. ulcerans*). ESX-5 is the exclusive ESX gene cluster found in slow-growing organisms and is absent in fast-growing species, underscoring its importance in pathogenicity and the slow-growing phenotype (Abdallah et al., 2008; Newton-Foot et al., 2016).

### 2.3 ESX-3 (ESAT-6 secretion system 3)

**Figure 2:**
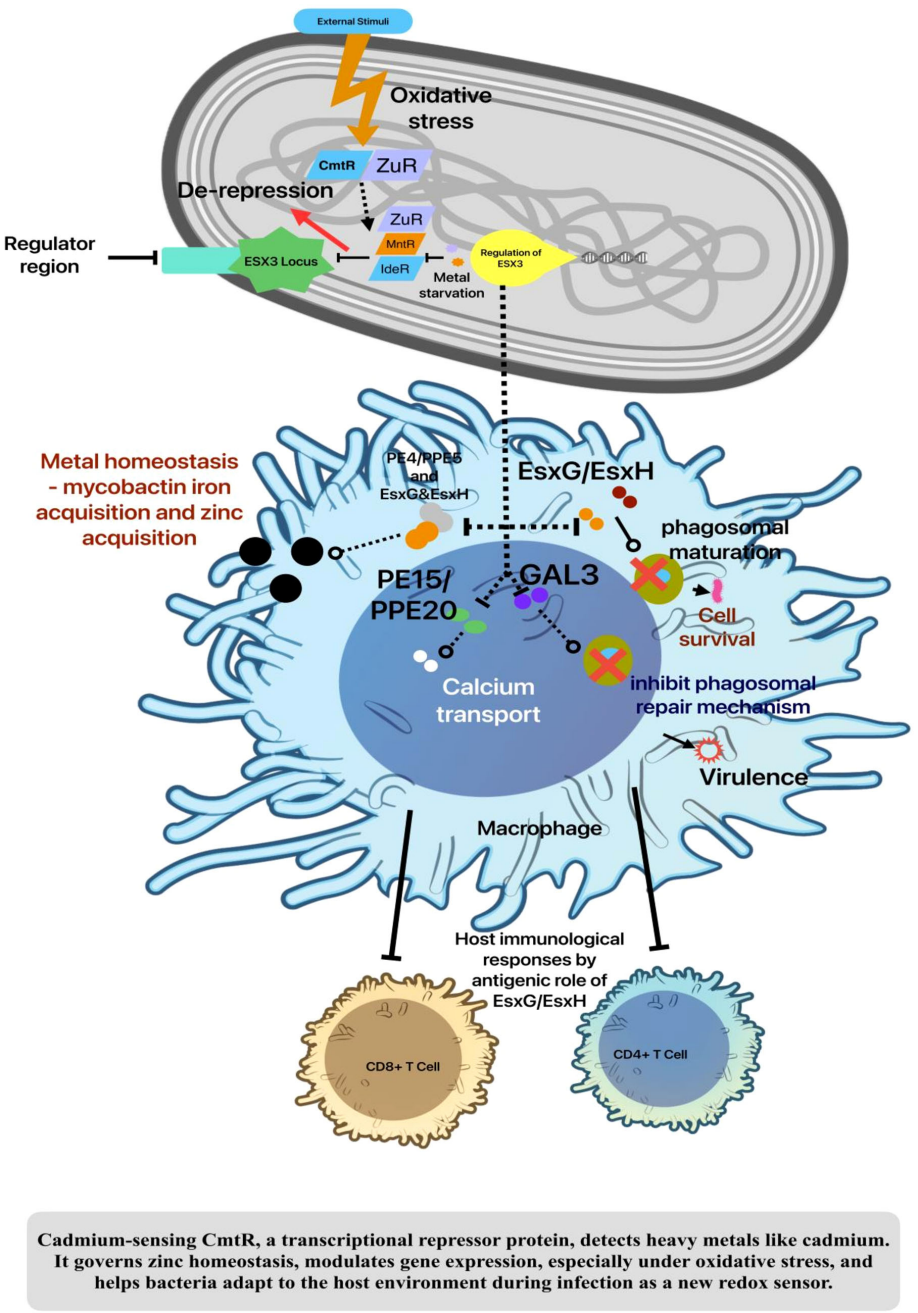
ESX-3 functions in MTB Metal acquisition.

ESX-3, the most conserved iron chelator in pathogenic and environmental mycobacteria, plays a role in the acquisition of iron and heme through mycobactin. However, ESX-3’s main function in bacteria is metal homeostasis. Mycobactin-bound iron is used by ESX-3. Additionally, the esx-3 gene cluster (*Rv0282–Rv0292*) is de-repressed by iron and zinc deficiency (Serafini et al., 2009) (**Figure 2**). Two promoters, one upstream of the cluster’s initial gene (*msmeg_0615*) and the other upstream of the esx genes (*msmeg_0620 and 0621*), control the expression of the esx-3 gene cluster in MTB and *M. smegmatis* (Rodriguez et al., 2002). *IdeR* regulates the first promoter in a way that is dependent on iron. The second promoter’s regulation mechanism remains unidentified (Maciag et al., 2009; Serafini et al., 2009; Tufariello et al., 2016; Fang et al., 2017). The pathogen MTB and the model organism *M. smegmatis* share 67% of the ESX-3 operon. The MTB has 4354 amino acids from ESX-3. In the hexamer model, EccC3 and EccB3’s transmembrane helices form a cavity that may host a substrate translocation pore in the multimer’s center. The hydrophobic transmembrane helices lack hydrated substrate conductors. A protein transit channel requires either a major transmembrane helical conformational change, possibly facilitate to *EccC3*, *EccD3*, and *EccE3* cytoplasmic domain movements, or a novel central pore transit mechanism (Poweleit et al., 2019). Besides, recent study revealed that ESX-3 heterotrimer composition: *PE5mt–PPE4mt–EspG3mm*. The *PE5mt–PPE4mt* heterodimer interacts with *EspG3* from different mycobacterial species to generate a mixed heterotrimer. Also, this study found 57% to 83% identity conservation in *EspG3*s, but residues interacting with *PPE4* were more conserved (Ekiert and Cox, 2014; Korotkova et al., 2014; Chen et al., 2017). The interaction of *EspG3* from different mycobacterial species with *PE5mt–PPE4mt* suggests species-invariant recognition by ESX systems. *PE5mt* and *PPE4mt* interact like *PE–PPE–EspG* heterotrimers (Chen et al., 2017; Williamson et al., 2020). The efflux mechanism among the numerous anti-TB medications, it has exhibited resistance to *Rv1258c*, which is part of the H+ antiporter-1 (DHA-1) subfamily of the Major Facilitator Superfamily (MFS). The supplementary analysis of the study indicates that the exclusive consequence of *Rv1258c* knockout is the down-regulation of ESX-3 among the five type VII secretion systems (Liu et al., 2019; Sun et al., 2024). Besides, the adaptation of bacteria to oxidative stress is associated with the regulation of Zn^2+^ levels. Some intracellular pathogens must adapt to the zinc toxicity imposed by the host. Recent findings indicate that ESX-3 in MTB (**Table 2**) possesses a function related to zinc transport. Zur regulates the expression of the esx-3 operon to maintain zinc homeostasis in MTB (Serafini et al., 2013; Li et al., 2020).

**Table 2:** Functional action of various biomarkers in ESX-3, 5 in MTBc.

Study	Biological markers	Outcomes	Technical platform
(Poweleit et al., 2019)	EccB3	Protomers' EccB3 periplasmic domains' wide contact area stabilizes dimerization. Two EccB3 proteins' homology models attach to the periplasmic domain, but it's too weak to pinpoint interactions. Oligomerization relies on the periplasmic area since EccB3 interacts with most cross-protomers.	Atomic model building
(Williamson et al., 2020)	EspG3 in MTBc strains	The 1:1:1 complex formed by mixing the complete MTB heterotrimer (58.8 kDa) with the mixed heterotrimer containing M. marinum EspG3 (58.1 kDa) had experimental molecular weights of 56.2 and 54.6 kD. Co-purified M. marinum EspG3, M. tuberculosis PE4–PPE5, M. kansasii (MKAN_17015), M. smegmatis (MSMEG_0622), and M. hassiacum EspG3.	size-exclusion chromatography & MALS (multiangle light scattering).
(Sun et al., 2024)	ESX-3. H37RvΔRv1258c	EccA3, EccC3, EccD3, and EccE3 were downregulated except EccB3. Main purpose of ESX-3 is iron. H37RvΔRv1258c has higher iron concentration in culture medium than WT, but lower in lysed bacilli supernatant. Rv1258c may change ESX-3 iron absorption and MTB iron metabolism.	qRT-PCR), inductively coupled plasma mass spectrometry (ICP-MS).
(Izquierdo Lafuente et al., 2021)	ESX-5– + eccC5, CpnT	After complementation with a functional eccC5 gene copy (ESX-5– + eccC5), CpnT secretion in M. marinum is restored in the ESX-5 secretion mutant, confirming that the mechanism is essential.	Double colony blot assays
(Bunduc et al., 2023)	EccD5	EccD5's importance in membrane complex protomers shows it's essential for complex growth and stability. Other membrane components were unaffected by eccB5, eccC5, or eccE5 deletion. MycP1 and MycP5, connected to the ESX-1 and ESX-5 systems, respectively, stabilize their T7SS membrane complexes.	BN-PAGE examination

### 2.4 ESX-4 (ESAT-6 secretion system-4)

The ESX-4 is present in all mycobacterial genomes that have been sequenced, little is known about its function. Esx-4 has only been investigated in two mycobacterial species that grow quickly: *M. smegmatis* and *M. abscessus.* According to research, ESX-4 is necessary for the recipient strain of *M. smegmatis* to undergo conjugal DNA transfer. Also, the effective use of heme iron in both MTB heme consumption pathways depends on the ancestral ESX-4 T7SS activities. The *eccC4* mutant eventually attains wild-type growth levels, suggesting that ESX-4 is not necessary for heme consumption. In *M. marinum*, interactions between ESX-4 and other T7SS, including ESX-1 and ESX-5, have been observed (Wang et al., 2021; Sankey et al., 2023).

Besides, the ESX-4 encodes EccC, a FtsK/SpoIIIE protein, as well as EsxU and EsxT, WXG proteins found in the FtsK-WXG clusters of *S. aureus, L. monocytogenes*, and *B. subtilis*. ESX-4 encodes EccD, EccB, and MycP, along with components of the WXG-FtsK cluster. These proteins may transport proteins into and across the outer mycomembrane using a more intricate secretion mechanism. The identification of the ESX-4 cluster in actinobacteria lacking mycomembranes indicates that these gene clusters secrete independently of mycomembrane translocation. The unknown function of this gene cluster, along with its occurrence and preservation in mycobacteria and other actinobacteria, implies that ESX-4 is significant for bacterial metabolism (Brodin et al., 2006; Newton-Foot et al., 2016).

### 2.5 ESX-5 (ESAT-6 secretion system-5)

ESX-5 secretion is crucial for membrane permeability and nutrient acquisition. The glycine-rich and repetitive *PE_PGRS* proteins, a significant subset of ESX-5 substrates, are linked to immune evasion and virulence. The precise role of ESX-5 and its substrates remains unclear (Ates et al., 2015). However, it releases most *PE* and *PPE* proteins and other substrates that modulate and evade the immune system. Notably, the locus-encoded PE/PPE proteins help ESX-5 secrete heterodimers. Similarly, recent research about ESX-1 in *M. marinum* shows that *EsxA* secretion requires *PPE68* co-secretion from the same gene cluster. Cell surface *PPE68* degradation occurred after export. Thus, findings suggest that Esx and PE/PPE proteins are specifically paired for co-secretion since *PE31/PPE18* did not complement Esx pair secretion. ESX-5 secretes PPE18-HA using *EsxM/EsxN* and PE/PPE proteins, proving that both substrate classes are necessary (Bunduc et al., 2023).

Furthermore, the *PE25–PPE41* dimer that interacts with EspG5mtu doesn’t seem to be suited for substrate binding. There are notable variations in the β2–β3 loop’s length and conformation. The loop, which stretches to 23 amino acid residues (Gly92-Asn114) in the *EspG5mtu*-*PE25–PPE41* structure (PDB ID 4KXR), has a significant interaction with the dimer. Twelve amino acid residues (Ser87-Leu98) make up the loop in the EspG3msm structure (PDB ID 4L4W). EspG5mtu’s conformational changes caused by *PE–PPE* binding may explain the observed structural variations, suggesting that EspG5’s interaction with *PPE41* in the *EspG5mtu-PE25–PPE41* structure differs from EspG1 and EspG3’s binding to their respective PPE protein (Korotkova et al., 2014; Tuukkanen et al., 2019). PE/PPE heterodimers’ secretion appears to be closely linked to Esx substrates. They may bind sequentially or simultaneously to activate EccC’s three ATPase domains, allowing membrane complex transport. The secretion dependence of the Esx pair on the PE/PPE pair and the need for equal expression levels of both heterodimers would be explained by this model. Moreover, the linker 2 domain of *EccC5* has a species-specific role in the secretion of *PE_PGRS* proteins, which constitute a significant subset of PE proteins secreted via ESX-5 (Damen et al., 2020). Notably, the ESX system responsible for the secretion of *CpnT* demonstrates that ESX-5 is capable of secreting the MTB toxin. Complementation reinstated *CpnT* secretion in the culture supernatant of the ESX-5-deficient strain. Infected macrophages failed to release *CpnT*. The findings indicate that *CpnT* functions as a substrate for ESX-5. The discovery that *CpnT*, a new toxin, is an ESX-5 substrate supports the previous finding that *M. marinum* and MTB ESX-5 mutants reduced macrophage cell death (Izquierdo Lafuente et al., 2021). Thus, further research on toxins and their correlation with the ESX is crucial to determine the MTB virulence.

### 2.6 Transcriptomic analysis of ESAT-6 secretion system

Recent study used proteomics and genome-wide transcriptional sequencing to examine the MH-S cells treated with His-EsxB or His-PPE68. By comparing the His-EsxB/His-PPE68 treated group to control samples, 159 out of 439 differentially expressed proteins (DEPs) were found, providing a thorough analysis of the host proteome. DEPs and differentially expressed genes (DEGs) were examined using KEGG pathway analysis in order to identify the primary signaling pathways implicated in host responses to His-EsxB/His-PPE68 therapy. The His-EsxB-treated group had 32 KEGG pathways with differentially expressed proteins based on Guo Y et al. study analysis, and the NCBI BioProject database has the raw data deposited under accession number PRJNA946337 (Guo et al., 2023). Noticeably, the MtZ strain’s exponential growth phase resulted in an almost twofold upregulation of both the *phoP* gene and the *Rv2034* gene, which positively controls *phoP* transcription. This implies that MtZ may become more virulent due to the phoP-ESX-1 system. During the stationary phase of H37Rv, MtZ has downregulated the *espA-espC-espD* genes, an operon thought to be a pathogenicity island (Comín et al., 2023).

The espA operon is one of the many esx-1-associated genes that are positively regulated by the transcriptional regulator *PhoP*, which is a component of the two-component system *PhoPR. PhoP* is regulated by pH, which suggests that the phagosome’s acidic environment triggers the esx-1 gene’s transcription, allowing bacteria to escape. Additionally, the *EspN* plays a critical role in infection by acting as an ESX-1 transcriptional regulator. In the recent study found that *EspN* and *EspM* act as a switch that controls the transcription of the ESX-1 gene. *EspM* inhibits *whiB6*, components of ESX-1 (including *EccA*), and substrate genes in the absence of the ESX-1 system, thereby preventing substrate accumulation due to lack of secretion. Similarly, another study determined that *PhoP*, *WhiB6*, and *EspR* regulate the expression of *esxB-esxA*; however, only *WhiB6* appears to directly influence the transcription of *esxB-esxA*, likely through binding to the promoter located upstream of *PE35* (Abdallah et al., 2019; Sanchez et al., 2020; Nicholson et al., 2024; Peters et al., 2024). Notably, numerous miRNAs, such as *miR-155, miR-1et7f*, and *miR-148a*, have been demonstrated to react to mycobacterial infection in an EsxA-dependent manner. Importantly, recent study elucidates the function of *miR-147*, a negative regulator of proinflammatory responses, in *M. marinum* infection. The ESX-1 system negatively regulates *miR-147*. Moreover, the increase of *miR-147* results in the inhibition of the TLR4/NF-κB signaling pathway, the intracellular persistence of *M. marinum*, and the synthesis of IL-6 and IL-10 (Kumar et al., 2015; Wu et al., 2019; Zuo et al., 2020).

## 3 Discussion

In this review, we analyzed the type VII secretion system, which consists of ESXs and functions among all ESX-1, 3, and 5, whereas these facilitate the translocation of proteins belonging to the WXG100 superfamily, characterized by a common two-helix hairpin structure that may form homo- or heterodimers. Additionally, a proteomic analysis found that ESX-3 secretes EsxG, EsxH, PE5–PPE4, PE15–PPE20, and possibly Rv2477c. Due to its lack of the YxxxD/E T7SS motif and reduced CF from ΔmbtB, Rv2477c is unlikely to represent a direct ESX-3 substrate ESX-1 and ESX-5 secrete several Esp substrates and PE–PPE family members, but Mtb ESX-3 secretes less (Rodriguez et al., 2002; Simeone et al., 2015). Notably, MTB encodes immune-modulatory proteins from the PE and PPE superfamilies. The PE/PPE protein superfamily’s benefit to survival under several stresses and pathologies is unknown. Previous research showed that PPE63 (*Rv3539*) was extracellular and has a C-terminal esterase extension. Thus, these proteins cannot be completely eliminated from the host’s immune response. By producing PPE63 in *M. smegmatis*, a non-pathogenic strain innately deficient in PPE63, its physiological role was shown. PPE63-expressing recombinant *M. smegmatis* affected colony shape, lipid content, and cell wall integrity (Anand and Kaur, 2023). Many MTB PPE proteins exhibit a reliance on the ESX-1, ESX-3, and ESX-5 systems. Conversely, the least studied are ESX-2 and ESX-4, whose functions remain unidentified. Phylogenetic analysis indicates that all other ESX systems originated from genetic duplication of ESX-4, the most primitive T7SS (Dumas et al., 2016; Izquierdo Lafuente et al., 2021). In this context, the role of STING in Mtb-induced IFN-β and Irg1 production in macrophages emphasizes the significance of the cGAS/STING/TBK-1/IRF-3 pathway for cytosolic DNA recognition. This mechanism requires the bacterial virulence factor ESX-1 to permeabilize the phagosome membrane and release bacterial DNA into the cytosol. Macrophage Irg1 and Type I IFN production was not stimulated by BCG without ESX-1, and STING had no effect on Irg-1 stimulation. Nevertheless, Irg1 is highly activated by MTB infection, producing itaconate to lower bacterial growth and neutrophilic inflammation (Bomfim et al., 2022).

Specifically, the role of ESX-1 in mediating the permeabilization and rupture of the host macrophage phagosomal membrane, which permits MTB to escape into the cytosol, is determined by various ESX-1-associated elements, including the translocation of *EspK* encoded by MMAR_5455 from the cytosol of *M. marinum* to the capsular layer and extracellular environment via ESX-1. Deficient in *EspK M. marinum* fully inhibits *EspB* secretion and diminishes *EsxA* and *EsxB* secretion, although to a lesser extent than isogenic EccCb1- or EccD1-deficient mutants. *M. marinum’s* deficiency in *EspK* resulted in reduced macrophage type I interferon responses, diminished virulence, and decreased secretion of *EsxA*, while *EsxB* secretion remained unaffected (Bosserman et al., 2019; Lienard et al., 2020; Lim et al., 2022). Bosserman et al. claim that the ESX-1 system secretes proteins that, through an unidentified mechanism, promote phagosomal lysis within the macrophage, but the recent study revealed that when espFMM was expressed in the WT strain, *whiB6* expression levels increased. These data support *EspE* and *EspF* operating independently. However, *WhiB6* autoregulation increases when *EspE* and *EspF* levels are low due to active secretion. As *WhiB6* levels grow, ESX-1 substrate production rises. *WhiB6* autoregulation is suppressed when *EspE* and *EspF* levels are high due to excessive production or decreased secretion, which reduces ESX-1 substrate production. When combined, these allow real-time substrate-level regulation and prevent cell substrate depletion. Thus, *EspE* and *EspF* improve ESX-1-mediated lysis and pathogenicity in these conditions (Bosserman et al., 2017; Chirakos et al., 2020; Sanchez et al., 2020; Wang et al., 2020).

Furthermore, the absence of *EsxA/EsxB* affects the secretion of several Esp proteins, complicating the exact function of each ESX-1 substrate. *EspC*, a crucial protein for *EsxA* secretion, is encoded by the espACD cluster, which is located more than 260 kb upstream of the esx-1 locus. Despite their smaller genomes, pathogenic mycobacteria like *Mycobacterium leprae* contain the highly conserved espACD locus. It’s a filamentous structure in the MTB cell envelope and an essential protein for ESX-1-secreted *EsxA* (Lou et al., 2017; Guo et al., 2021). In MTB-infected mice, neutrophils and monocyte-derived cells are the main lung tissue reservoirs, and ESX-1 boosts granuloma core CXCL1, 2, and 5 synthesis and neutrophil accumulation. However, neutrophils are necessary for ESX-1-mediated pathology, suggesting that neutrophils are host-detrimental in mycobacterial infections and play a previously unknown role in ESX-1-dependent virulence (Cohen et al., 2018; Lienard et al., 2023). In contrast, the *EsxA/EsxB* expression is also needed to kill macrophages uptake-independently by extracellular bacterial aggregates. However, deleting *espA* has little effect on macrophage killing when *EsxA/EsxB* secretion is eliminated unless the ESX-1-secreted *EspB* protein is missing. Moreover, *EsxA/EsxB* expression is required to secrete the cleaved 50 kDa *EspB* isoform, but not the full-length 60 kDa isoform. For *EspB* to interact with phospholipids, oligomerize into channel-shaped heptamers, and be fully virulent, *MycP1* must cleave it. Importantly, the N-terminal domain of the full-length oligomeric *EspB*’s cryo-EM structure formed a heptameric ring. The *EspB* heptamer’s inner diameter was unexpectedly near to 4.5 nm, suggesting that the core channel might carry heterodimeric ESX-1 substrates like ESAT6-CFP10 (Piton et al., 2020; Lim et al., 2022; Sengupta et al., 2023; Toniolo et al., 2023).

ESAT-6 reduces MTB H37Ra-induced apoptosis in THP-1(A) macrophages via TLR2. Bioinformatics analysis shows that ESAT-6 reduces macrophage apoptosis via the Caspase-9/Caspase-3 pathway, as shown by Western blotting. ESAT-6, a virulence factor from the RD1 region of aggressive MTB strains, induces host cell death. Due to the absence of affinity tags, “native” ESAT-6 from MTB cells is acquired more strictly than recombinant ESAT-6. Despite not being detected in MTB infection, N-terminal acetylation explains the differences between recombinant and native ESAT-6. However, N-acetylation increases ESAT-6 release from CFP-10 under acidic environments. ESAT-6 inhibits MTB-induced Caspase-9 and Caspase-3 at the appropriate dose. ESAT-6-releasing MTB strains may reduce macrophage apoptosis more than attenuated strains. High ESAT-6 levels may trigger THP-1(A) macrophage apoptosis, explaining these differences. Validation investigations will compare the H37Rv-ΔRD1 strain, lacking the entire RD1 region encoding the ESAT-6 gene, to the wild-type H37Rv and the recombinant ESAT-6 used in the current study to confirm the importance of ESAT-6 in cellular immunity (Lin et al., 2019; Zhang et al., 2025).

The release of virulence factors by bacteria into host cells or the extracellular milieu is crucial for their survival. Consequently, the Type VII system has evolved; nevertheless, the T7SS is essential to determine the host immune evasion of MTB, and various studies have shown existing knowledge about ESX, but autophagy-regulated genes and correlation with the ESX system will lead to determining the survival of MTB. However, ESX-1 proteins may help Mtb-encoded factors enter the cytosol to block autophagy instead of ESAT-6. *SapM* suppresses BCG-containing phagosome formation, which may explain Mtb’s antiautophagic action. Shin et al. revealed that MTB-enhanced intracellular survival (Eis) factor in infected macrophages’ cytoplasm negatively influences murine macrophage autophagy induction. Although Eis’s role in infected DC is unknown, these data imply that Mtb hindered autophagy in numerous ways (Shin et al., 2010; Romagnoli et al., 2012).

Recent research used intracellularly expressed E11rv to regulate proliferation. E11rv had functional effects in the media and cytoplasm, suggesting that MTB communicates with different cellular compartments in the phagosome. ESAT-6 exhibits multiple functions, including apoptosis (Behura et al., 2019), interferon-γ suppression (Bates et al., 2024), autophagy inhibition (Dong et al., 2016; Behura et al., 2019), antigen presentation obstruction, type I interferon induction, and ESX-1-dependent Mtb antigen integration into MHC-I presentation via cytosolic processing. Most of these functions require ESAT-6 to enter the cytoplasm, either complete phagosomal escape of MTB followed by ESAT-6 secretion, or phagosomal membrane disruptions that allow bidirectional compartment communication (Behura et al., 2019).

Recent research demonstrated that recombinant EsxA does not lyse WI-26 and A549 lung epithelial cells. Conrad et al. showed that membrane permeabilization does not require acidity by maintaining ESX-1-mediated phagosomal permeabilization with bafilomycin (Conrad et al., 2017). Lienard et al. found that infected macrophages with Mm ESX-1 transposon mutants, including those that disrupt EsxA secretion, and showed that EsxAB-independent processes determine cytosolic translocation. During infection, mycobacteria tear phagosome membranes, but EsxA’s pH-dependent membrane-permeabilizing function in liposomal membranes is unknown. Studying mycobacteria and host cell properties such target membrane properties is essential and way forward to future investigation (Lienard et al., 2020). Also, identifying the role of *PE-PGRS* proteins and conducting a transcriptomic analysis of these regulated genes will help determine the pathophysiology of the bacteria (Aguilera et al., 2020; Yu et al., 2021; Collars et al., 2023; Aghoram et al., 2025).

Recent studies revealed that BCG::ESX1Mtb was protected by an augmented T cell response to ESAT6 (EsxA), an immunodominant protein produced by the ESX1 locus of Mtb. Recently, the release of full-length ESAT6 stimulates cytosolic pattern recognition and protective non-specific immune responses against MTB. This study employed 24 vaccination regimens with three BCG strains (BCG, BCG::ESX1Mtb, and a derivative) together with eight delivery combinations in a mouse model of tuberculosis. ESX is a therapeutic tool for MTB (Kupz et al., 2016; Flores-Valdez et al., 2022; Aarthi et al., 2025).

## 4 Conclusion

This review analyses the functions of the ESX-1, -3, and -5 secretion systems in the pathogenesis of TB. The fundamental actions of specific antigenic structures and their associations with autophagy-regulated proteins, such as PE-PPE, are examined. The ESX system’s genes are linked to the phagosome membrane’s permeabilization, which permits the release of bacterial nucleic acids into the cytosol. Thus, the importance of the T7SS in bacterial survival needs more research is necessary to elucidate the unidentified functions associated with the ESX system. Consequently, the investigation of genes linked to the ESX system is essential for comprehending the virulence of MTB and the mechanisms governing autophagy-regulated functions.
